# Preclinical profiles of SKB264, a novel anti-TROP2 antibody conjugated to topoisomerase inhibitor, demonstrated promising antitumor efficacy compared to IMMU-132

**DOI:** 10.3389/fonc.2022.951589

**Published:** 2022-12-23

**Authors:** Yezhe Cheng, Xiaoxi Yuan, Qiang Tian, Xiuying Huang, Yang Chen, Yuzhi Pu, Hu Long, Mingyu Xu, Yafei Ji, Jia Xie, Yuping Tan, Xi Zhao, Hongmei Song

**Affiliations:** Center of Translational Medicine, Sichuan Kelun-Biotech Biopharmaceutical Co., Ltd., Chengdu, China

**Keywords:** antibody drug conjugate, SKB264, TROP2, PK-PD, solid tumor

## Abstract

**Purpose:**

The aim of this study was to improve the intratumoral accumulation of an antibody–drug conjugate (ADC) and minimize its off-target toxicity, SKB264, a novel anti-trophoblast antigen 2 (TROP2) ADC that was developed using 2-methylsulfonyl pyrimidine as the linker to conjugate its payload (KL610023), a belotecan-derivative topoisomerase I inhibitor. The preclinical pharmacologic profiles of SKB264 were assessed in this study.

**Methods:**

The *in vitro* and *in vivo* pharmacologic profiles of SKB264, including efficacy, pharmacokinetics–pharmacodynamics (PK-PD), safety, and tissue distribution, were investigated using TROP2-positive cell lines, cell-derived xenograft (CDX), patient-derived xenograft (PDX) models, and cynomolgus monkeys. Moreover, some profiles were compared with IMMU-132.

**Results:**

*In vitro*, SKB264 and SKB264 monoclonal antibody (mAb) had similar internalization abilities and binding affinities to TROP2. After cellular internalization, KL610023 was released and inhibited tumor cell survival. *In vivo*, SKB264 significantly inhibited tumor growth in a dose-dependent manner in both CDX and PDX models. After SKB264 administration, the serum or plasma concentration/exposure of SKB264 (conjugated ADC, number of payload units ≥1), total antibody (Tab, unconjugated and conjugated mAb regardless of the number of the payload units), and KL610023 in cynomolgus monkeys increased proportionally with increasing dosage from 1 to 10 mg/kg. The linker stability of SKB264 was significantly enhanced as shown by prolonged payload half-life *in vivo* (SKB264 *vs*. IMMU-132, 56.3 h *vs*. 15.5 h). At the same dose, SKB264’s exposure in tumor tissue was 4.6-fold higher than that of IMMU-132.

**Conclusions:**

Compared with IMMU-132, the longer half-life of SKB264 had a stronger targeting effect and better antitumor activity, suggesting the better therapeutic potential of SKB264 for treating TROP2-positive tumors.

## Introduction

TROP2, a trophoblast cell-surface antigen, also known as tumor-associated calcium signal transductor (TACSTD2) is overexpressed in a variety of human epithelial cancers, including breast, lung, gastric, colorectal, pancreatic, prostatic, cervical, head-and-neck, and ovarian carcinomas (1, 2). Elevated TROP2 expression is associated with tumor invasion, aggression, progression, and metastasis (3–5). Preclinical and clinical studies have demonstrated the efficacy of anti-TROP2 antibody–drug conjugates (ADCs), such as sacituzumab govitecan (IMMU-132), an anti-TROP2 humanized antibody-SN-38 conjugate for cancer treatment (6, 7). The U.S. Food and Drug Administration (FDA) and the European Medicines Agency (EMA) approved IMMU-132 as a first-in-class ADC for cancer treatment. The clinical results for the use of IMMU-132 to treat refractory solid tumors were quite encouraging (8). The objective response rate (ORR) of IMMU-132 in drug-resistant triple-negative breast cancer (TNBC) patients reached 33% and definitive efficacy was demonstrated in patients after immunosuppressive or chemotherapy (9, 10). The antibody–drug linker is an important feature of IMMU-132, because payload release depends on the acid-mediated cleavage of the carbonate linkage between the linker and SN-38 (11), by which SN-38 is continually released at the targeted tumor site (12). However, the half-life of IMMU-132 in serum is relatively short (~1 day), because of the less stable thiol–maleimide coupling due to the known retro-Michael addition (13). Breakage of the linker–payload conjugate from the maleimide-based ADC can occur through thiol exchange under physiological conditions, thus leading to off-target effects. Accordingly, IMMU-132 may have relatively high off-target effects.

SKB264 is a TROP2-targeting ADC that shares the same monoclonal antibody (mAb) as IMMU-132. The mAb is conjugated with seven to eight molecules of novel payload *via* the coupling sites on the reduced inter-chain disulfide bonds. TL033, a novel drug-linker intermediate, contains the toxic payload KL610023 (also named T030), which is a topoisomerase I inhibitor belotecan derivative with a bystander effect, and can arrest cell cycle at the G2/S stage after its internalization, leading to cell death (14). Moreover, the 2-methylsulfonyl pyrimidine has been used as a novel ADC coupling group between the linker and mAb through irreversible nucleophilic aromatic substitution, achieving higher ADC stability (compared to IMMU-132) due to the lack of retro-Michael reaction *in vivo* (15). Our previous studies showed that the structure of both the payload and linker contributed to the increased ADC stability, thus maintaining ADC bioactivity. The half-life of SKB264 was around 57 h in the tumor-bearing mice model, while that of IMMU-132 was 14 h in the same model (10). Comparing the molecular structure of linker between SKB264 and IMMU-132, they have the same benzyl carbonate site, which is considered a pH-mediated cleavage site for releasing the payload from the linker in the tumor microenvironment. With a longer half-life than IMMU-132, SKB264 might achieve a different and more effective balance of extracellular stability and intracellular rupture of the ADC, resulting in a better efficacy. Preclinical studies of SKB264 have demonstrated its excellent ability to inhibit tumor growth, such as mammary, lung, stomach, and colorectal cancers, in a variety of CDX and PDX models. However, the characteristics of SKB264 remain incompletely understood. Therefore, extensive studies on SKB264 are needed to achieve a better therapeutic index through improved dynamics of tissue distribution and payload release.

The present study evaluated the preclinical profiles of SKB264, related to efficacy, PK-PD, and safety, compared with those of IMMU-132. The results showed that SKB264 had a stronger targeting effect and excellent antitumor activity with favorable ADC stability and safety profiles, suggesting the potential clinical utility of SKB264 for treating cancers with TROP2 overexpression.

## Materials and methods

### Antibodies and ADCs

SKB264 mAb is a humanized anti-TROP2 mAb with reference to the same amino acid sequence as IMMU-132. SKB264 is a novel anti-TROP2 ADC, in which SKB264 mAb is conjugated with seven to eight molecules of novel payload named KL610023 *via* the coupling sites on the reduced inter-chain disulfide bonds. Human IgG1 (hIgG1), KL610023, and the linker of SKB264 are developed by Kelun-Biotech (Sichuan Chengdu, China). IMMU-132, a reference ADC used in this study, was purchased from Immunomedics, Inc. (Morris Plains, NJ).

### Cell lines

NCI-N87 (human gastric carcinoma), BxPC-3 (human pancreas adenocarcinoma), and Calu-3 (human non-small cell lung cancer) were purchased from ATCC (American Type Culture Collection). Both HCC1806 (human breast adenocarcinoma) and NCI-H23 (human non-small cell lung cancer) were purchased from CoBioer (Nanjing, China). The TROP2-overexpressing NCI-H23 (TROP2+) line, which is a subtype of NCI-H23 and constructed by Kelun-Biotech, and other cell lines, including NCI-N87, BxPC-3, HCC1806, and CI-H23 (parental), were cultured in RPMI 1640 medium supplemented with 10% fetal bovine serum (FBS). Calu-3 cells were cultured in Minimum Essential Medium (MEM) supplemented with 10% FBS and non-essential amino acids. All cell lines were maintained at 37°C in 5% CO_2_. All the cell lines were examined by short tandem repeat authentication *via* Genegle (Chengdu, China) and tested for mycoplasma using a mycoplasma detection kit (Yise, Shanghai, China).

### TROP2 expression in tumor cell lines

Flow cytometry (FCM) was used to detect TROP2 expression in tumor cell lines. First, cells were incubated with the following antibodies on ice for 30 min: 1 μg/ml phycoerythrin (PE)-conjugated mouse IgG2a, k isotype control, or PE-conjugated anti-human TROP2 antibody (eBioscience, San Diego, CA). After rinsing, the labeled cells were analyzed by FCM (BD Accuri C6). The following gating strategy was used for FCM. In the FSC/SSC plot, cells were selected using a gate for high FSC cells except for cell debris. Then, single cells were selected using a gate for a single slope in the FSS-A/FSC-H plot. In the PE-A plot, nonspecific binding was excluded by isotype using a gate with little PE-A cells.

### Binding and dynamic affinity

An enzyme-linked immunosorbent assay (ELISA) was used to evaluate the binding and dynamics affinity. Human TROP2 protein (1 μg/ml) (Sino Biological Inc, Wayne, PA) was coated on a 96-well plate overnight at 4°C. The plate was then blocked by Super block reagent (ScyTek Inc. Logan UT) at room temperature for 1 h. Diluted SKB264, SKB264 mAb, or isotype control (human IgG1 antibody) was added for incubation at 37°C for 1 h. After washing, the diluted (1:10,000) goat anti-human IgG Fc-horseradish peroxidase (HRP) (Abcam, Waltham, MA) was added for further incubation for 45 min at 37°C. Finally, TMB solution was added for absorbance measurement at 450 nm using a SpectraMax Plus384 Microplate reader (Molecular Devices, USA).

Surface plasmon resonance (SPR) was an alternative method to evaluate dynamic affinity, in which human TROP2 protein was captured with goat anti-human IgG Ab immobilized onto a CM5 sensor chip (Biacore Inc., Raleigh, NC). Serial titrations of SKB264 or IMMU-132 ranging from 0.0823 to 2.22 nM and running buffer were individually injected over the surface, which was captured human TROP2 for an association phase of 120 s, followed by a 600-s dissociation time. Kinetic parameters were calculated using the BIA evaluation software with the average values of two datasets collected on separate days. Association (*K*_a_) and dissociation (*K*_d_) rate constant values were calculated by fitting analysis assuming a Langmuir binding model (16) and a stoichiometry of (1:1) to determine *K*_D_ (equilibrium dissociation constant) values.

### Cytotoxic assay

Tumor cells were seeded on 96-well plates at the following concentrations: HCC1806 (3,000 cells per well), NCI-N87 (5,000 cells per well), BxPC-3 (2,000 cells per well), Calu-3 (8,000 cells per well), NCI-H23 (TROP2+, 3,000 cells per well), and NCI-H23 (parental, 3,000 cells per well). After overnight incubation, the diluted testing items were added respectively. After 72 h, cell viability was evaluated using a CellTiter-Glo Luminescent Cell Viability Assay (Promega Corp. Madison, WI), following the manufacturer’s instruction.

### CDX models

All animal experiments in this study were conducted in the laboratory fully accredited by the Association for Assessment and Accreditation of Laboratory Animal Care (AAALAC) International. The contents and procedures related to animal experiments in the study complied with the relevant laws and regulations in the experimental animal use and management and the relevant requirements of the Institutional Animal Care and Use Committee (IACUC). The study protocol was approved by IACUC (IACUC Serial Number: IACUC-A2018086-T014-01).

The CDX models of HCC1806 (breast cancer) and NCI-N87 (gastric carcinoma) cells were developed by subcutaneously engrafting tumor cells into female BALB/c nude mice (GemPharma, Nanjing, China). When the average tumor volume reached about 120 mm^3^, mice were randomized into five groups (*n* = 8) and subsequently administered by intravenous (i.v.) injection with the testing item twice a week for six times. SKB264 treatments were performed at doses of 1, 3, and 10 mg/kg in the HCC1806 xenograft model and 0.3, 1, and 3 mg/kg in the NCI-N87 xenograft model. Irinotecan (30 mg/kg) treatment was an arm of the standard of care in the CDX models. The percentage of tumor growth inhibition (TGI) was defined as %TGI = [1 − (*T*_i_ − *T*_0_)/(*V*_i_ − *V*_0_)] × 100%, to evaluate the antitumor efficacy *in vivo*, where *T*_0_ and *T*_i_ were the mean tumor volume of the treatment group on the days of randomization and measurement, respectively, and *V*_0_ and *V*_i_ were the mean tumor volume of the control group on the days of randomization and measurement, respectively. Body weight changes and mortality were recorded to evaluate the tolerability of the testing items.

### PDX models

The HuPrime^®^ breast cancer xenograft model, which utilized primary BR1282 breast cancer derived from a female patient, was provided by Crown Bioscience Inc. (Cambridge, MA), and four gastric cancer PDX models, namely, 0406022, A11068, 0501116, and A19058, were provided by Personal Oncology (Nanjing, China). The BR1282 PDX model was confirmed to be TROP2-positive (high expression) by pathological examination. The four gastric cancer PDX models (0406022, A11068, 0501116, and A19058) were also confirmed to have high, moderate, low, and negative TROP2 expression, respectively. Immunohistochemistry (IHC) analysis of the paraffin-embedded PDX tissue section was performed with anti-TROP2 antibody (Abcam, Waltham, MA), following the set procedure of the Bond RX Fully Automated Research Stainer (Leica Biosystems Inc., Buffalo Grove, IL). NDP2.0-HT, a next-generation viewer software (Hamamatsu), was used for the image analysis. After primary tumor tissue harvested in “stock mice”, the tumor fragments (2 to 3 mm in length) were implanted subcutaneously into the right flank of BALB/c nude (BR1282) or NCG (four gastric cancer PDX models) mice purchased from GemPharma (Nanjing, China). Mice were randomized in five groups when the average tumor volume reached about 80–120 mm^3^ (*n* = 8 or 6). The tumor-bearing mice were treated with SKB264 *via* i.v. injection at doses of 0.5, 1.5, and 5 mg/kg for BR1282 and 1, 3, and 10 mg/kg for gastric cancer PDX models twice a week for six times. Irinotecan, paclitaxel, or paclitaxel (albumin-bound form) were the standard of care in the PDX models. Tumor volume and body weight were measured twice a week. Tumor weight was measured at study termination. Body weight changes and mortality were recorded to evaluate the tolerability of the testing items.

### PK properties of SKB264 and KL610023 in cynomolgus monkeys

The PK properties of SKB264 and Tab were determined by ELISA, for which the lower limit of quantitation was 7.813 ng/ml. The serum concentration of KL610023 was measured by a validated liquid chromatography tandem mass spectrometry (LC/MS-MS), for which the lower limit of quantitation was 0.100 ng/ml. A total of 24 cynomolgus monkeys were randomized into four groups (*n* = 3/sex/group). Groups #1, #2, and #3 were treated with 1, 3, and 10 mg/kg of SKB264, respectively, *via* 15* min* i.v. infusion once. Group #4 received 0.24 mg/kg of unconjugated KL610023 *via* i.v. infusion once. The serum concentrations of SKB264, Tab, and KL610023 were continually measured up to 672 h post-dose.

### Tissue distribution of SKB264 after single i.v. injection in tumor-bearing nude mice

A total of 96 female nude mice bearing human HCC1806 tumor cells were divided into 16 groups (*n* = 6 mice/group) according to body weights and tumor volumes. After receiving a single i.v. injection of [^3^H]KL610023, at 0.083, 4, 24, and 72 h, as well as a single i.v. injection of [^3^H-KL610023]SKB264, [^3^H]SKB264-mAb, or [^3^H-mAb]SKB264 at 1, 24, 168, and 336 h post-dosing, blood, tumor tissue, and 16 types of normal tissues were collected from the euthanized mice.

Plasma samples were directly quantified by liquid scintillation counting (LSC) without further manipulation; however, the blood and tissue samples were digested with 1N of KOH at 90°C prior to quantification by LSC. The tissue residue approach (TRA) for blood, plasma, and tissues was converted to nanogram equivalent of [^3^H]KL610023 or TRA (ng Eq./g).

### Toxicity assessment in cynomolgus monkeys

Forty cynomolgus monkeys were randomized into four groups (*n* = 5 animals/sex/group). The experimental animals were administered with 0.9% NaCl injection (negative control) or SKB264 (25, 50, or 75 mg/kg) *via* i.v. infusion once every 2 weeks for four doses (**Table 1**). The i.v. infusion volume of the SKB264 or the control was 10 ml/kg. Toxicity assessments were as follows: mortality/moribundity, clinical signs, body weights, food consumption, body temperature, ophthalmic examinations, local tolerance (injection site) evaluations, safety pharmacology [12-lead electrocardiogram (ECG), blood pressure, heart rate, and respiration examinations], clinical pathology (hematology, coagulation, and serum chemistry), urinalysis, cytokine levels [interleukin-2 (IL-2), interleukin-6 (IL-6), interleukin-8 (IL-8), tumor necrosis factor-alpha (TNF-α), and interferon-gamma (IFN-γ)], bone marrow smear evaluations, toxicokinetics (TK), immunology, anti-drug antibody (ADA) analysis, organ weights, and macroscopic and microscopic examinations.

**Table 1:** Summary results of repeated SKB264 administration in cynomolgus monkeys.

Category	Details
Animal species	Cynomolgus monkeys
Dose	0, 25, 50, 75 mg/kg
Dosing frequency	Once every 2 weeks, four times in total
Number of animals	5/sex/group
Dead/moribund animals	75 mg/kg: two females and three males were moribund or found dead ≤50 mg/kg: No abnormality
Body weight	75 mg/kg: Decrease in body weight was observed in the surviving females, and no significant abnormality was noted in the body weight of the surviving males ≤50 mg/kg: No abnormality
Hematology	≥50 mg/kg: Decreases in WBC (NEU, LYM), RBC, HGB, HCT, RET, and RET% 25 mg/kg group: Decreases in RBC, HGB, HCT, RET, and RET%, but only lower RET and RET% were found in the males.
Toxic target organs/tissues	≥50 mg/kg: jejunum, ileum, cecum, skin, sternal bone marrow, and vagina ≥25 mg/kg: thymus
HNSTD	50 mg/kg

### Comparison of *in vivo* efficacy and PK-PD between SKB264 and IMMU-132

Female BALB/c nude mice (Charles River, Beijing, China) were inoculated with 2×10^6^ HCC1806 cells in the right front flank. To evaluate *in vivo* efficacy of ADC, when the average tumor size reached about 120 mm^3^, mice were randomized into 7 groups (*n* = 6 mice/group). The mice were treated with SKB264 or IMMU-132 at doses of 1, 3, and 10 mg/kg twice a week for six times *via* i.v. injection. To evaluate the PK-PD of ADC, when the average tumor size reached approximately 270 mm^3^, mice were randomized into three groups (*n* = 5/time point). The mice received a single i.v. injection of control hIgG1, SKB264, or IMMU-132 (3 mg/kg), respectively. Blood samples (from five mice) were collected at 1 h, 4 h, 8 h, 24 h, 48 h, 96 h, 192 h, or 336 h post-dosing to detect the concentration of payload at each time point. The rate of TGI (*n* = 5 mice, measured at endpoint, i.e., 336 h) was determined to evaluate the antitumor efficacy of ADC. Body weight changes and mortality were also recorded to evaluate the tolerability of the testing items.

### *In vitro* stability of SKB264 and IMMU-132 in monkey and human plasma

To evaluate the *in vitro* stability of SKB264 and IMMU-132 in monkey and human plasma, the release rate of payload from SKB264 and IMMU-132 at the concentration of 50 μg/ml at 37°C up to 144 h was examined by measurement of payload concentration in plasma using LC/MS-MS, with a lower limit of quantitation was 2.00 ng/ml.

### Statistical analysis

Phoenix WinNonlin 8.1 was used to calculate the PK parameters in the non-compartment model, including *t*_1/2_, *T*_max_, *C*_max_, AUC_0-t_, and AUC_0-∞_. The *in vitro* data were analyzed and displayed using software PRISM 5.0 (GraphPad). The half maximal effective concentration (EC_50_) and half maximal inhibitory concentration (IC_50_) were calculated using a nonlinear regression model with sigmoidal fitting. The *in vitro* antitumor activities of drugs were evaluated by a two-tailed paired *t*-test.

## Results

### Structure of SKB264

**Figure 1:**
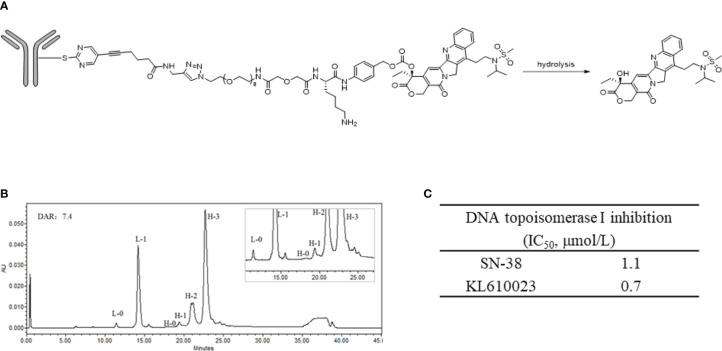
Structure and characterization of SKB264. **(A)** The chemical structure of the linkage of SKB264. **(B)** The distribution of the conjugated drug determined by the reverse-phase chromatography analysis (L-0: Unconjugated light chain, L-1: Light chain conjugated with one payload, H-0: Unconjugated heavy chain, H-1: Heavy chain conjugated with one payload, H-2: Heavy chain conjugated with two payloads, H-3: Heavy chain conjugated with three payloads). **(C)** Comparison of the inhibitory activity of cell-free DNA topoisomerase I between SN-38 and KL610023.

The structure of SKB264 is shown in **Figure 1A**. SKB264 is a TROP2-targeting ADC, which consists of a recombinant humanized anti-TROP2 antibody and a topoisomerase I inhibitor derived from Belotecan (KL610023), integrated together *via* a sulfonyl pyrimidine-CL2A-carbonate linker. The payload is conjugated with the antibody through irreversible nucleophilic aromatic substitution by tris (2-carboxyethyl) phosphine hydrochloride (TCEP HCl), a reducing agent that can reduce the interchain disulfide bounds. The drug-to-antibody ratio (DAR) of SKB264 was approximately 7.4, which was determined by the reversed-phase chromatography (RPC), and might be the theoretical maximum drug loading number for conventional interchain cysteine conjugation (**Figure 1B**). SKB264 was designed to release payload KL610023 after internalization by TROP2-expressing tumor cells. The inhibitory effect of KL610023 on topoisomerase I was almost similar to that of SN-38, as measured by a topoisomerase I-mediated DNA relaxation assay (**Figure 1C**).

### Action mechanism of SKB264

**Figure 2:**
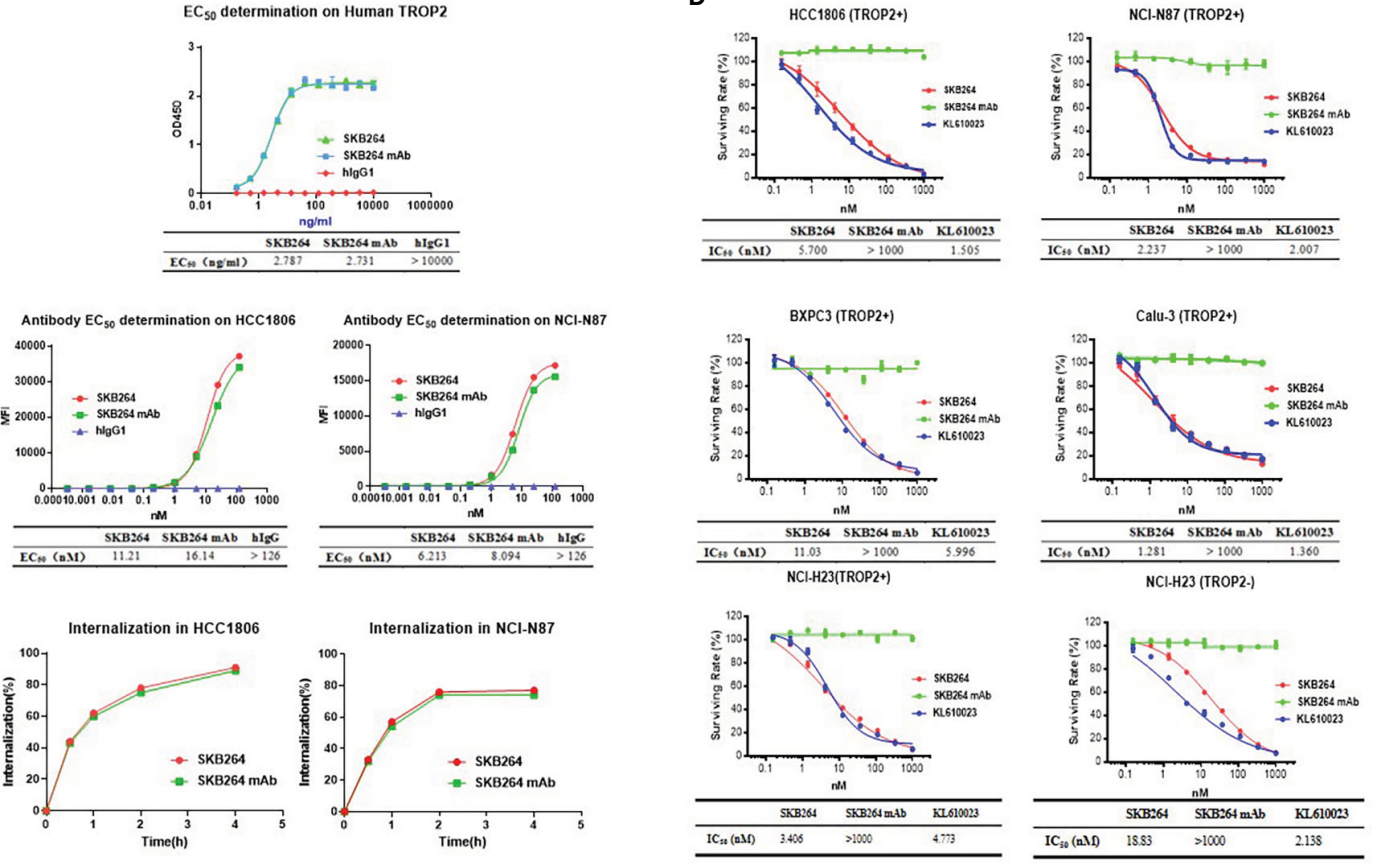
*In vitro* activities of SKB264. **(A)** Binding to human TROP2 estimated by ELISA (duplicates, mean ± SEM). **(B)** SKB264 binding to HCC1806 and NCI-N87 estimated by FACS. **(C)** Internalization of SKB264 or SKB264 mAb in HCC1806 and NCI-N87 cells. **(D)** *In vitro* cell growth inhibitory activity (triplicates, mean ± SEM) in HCC1806, NCI-N87, BxPC-3, Calu-3, NCI-H23 (TROP2+), and NCI-H23 (parental) cells.

To examine whether the anti-TROP2 mAb plays a targeting role *via* specifically recognizing the highly expressed TROP2 on the surface of tumor cells, the following experiments were conducted (17). At the protein level, both SKB264 and SKB264 mAb had similar affinity to TROP2 protein, with an EC_50_ of 2.787 ng/ml and 2.731 ng/ml, respectively (**Figure 2A**), indicating that coupling payload KL610023 does not affect the affinity of mAb terminal. Compared to SKB264 mAb, the cell-level affinity of SKB264 showed a similar binding ability to TROP2-positive cells HCC1806 and NCI-N87. The EC_50_ of SKB264 was 11.21 nM and 6.213 nM, respectively, in two cell lines as mentioned above, while the EC_50_ of SKB264 mAb in them was 16.14 nM and 8.094 nM, respectively (**Figure 2B**). Those results suggest that the targeting role of SKB264 is mainly dependent on anti-TROP2 antibody, which can specifically bind to TROP2-positive cells, as KL610023 did not affect mAb affinity with TROP2. The results of the endocytosis experiment also showed that the percentage of endocytosis of SKB264 and SKB264 mAb was increased over time in TROP2-positive cells (HCC1806 and NCI-N87, **Figure 2C**). Taken together, these data imply that SKB264 specifically binds to TROP2-positive cells by targeting the TROP2 protein expressed on the cell surface and thereby inducing endocytosis.

### *In vitro* antitumor activity of SKB264

**Figure 3:**
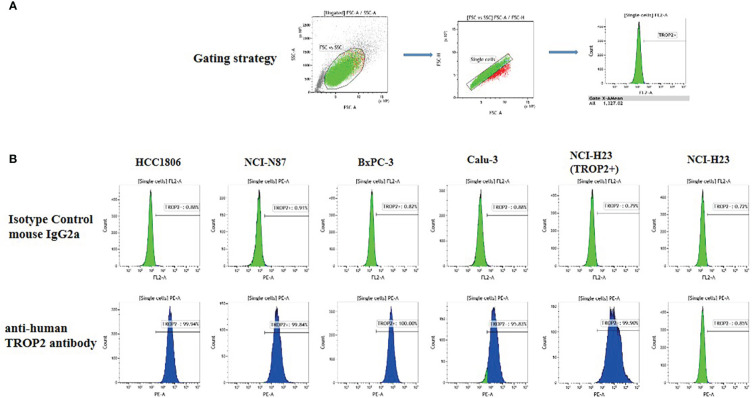
TROP2 expression on the cell surface. **(A)** Gating strategy. **(B)** FACS measurement results for six cell lines, namely, HCC1806, NCI-N87, BxPC-3, Calu-3, NCI-H23 (TROP2+), and NCI-H23 (parental).

To evaluate the effect of SKB264 on tumor cell proliferation, *in vitro* growth inhibition of cancer cell lines was determined by CellTiter-Glo Luminescent Cell Viability Assay. Cancer cell lines, including HCC1806, NCI-N87, BxPC3, Calu3, and NCI-H23 (TROP2+), highly expressed TROP2, while NCI-H23 (parental) was TROP2-negative (**Figure 3**). As shown in **Figure 2D**, unlike SKB264 mAb alone that has no inhibitory effect on tumor proliferation, both SKB264 and KL610023 could inhibit tumor cell growth, with IC_50_ values of 1.281–18.83 nM (SKB264) and 1.360–5.996 nM (KL610023). NCI-H23 (TROP2+) cells were more sensitive to SKB264 than parental cells (TROP2−). These results indicate that SKB264 exhibits tumor suppression ability mainly through its payload terminal. Taken together, after SKB264 endocytosed through its interaction with TROP2 on cell surface, the releasing free KL610023 can inhibit tumor cell proliferation.

### *In vivo* antitumor activity of SKB264

**Figure 4:**
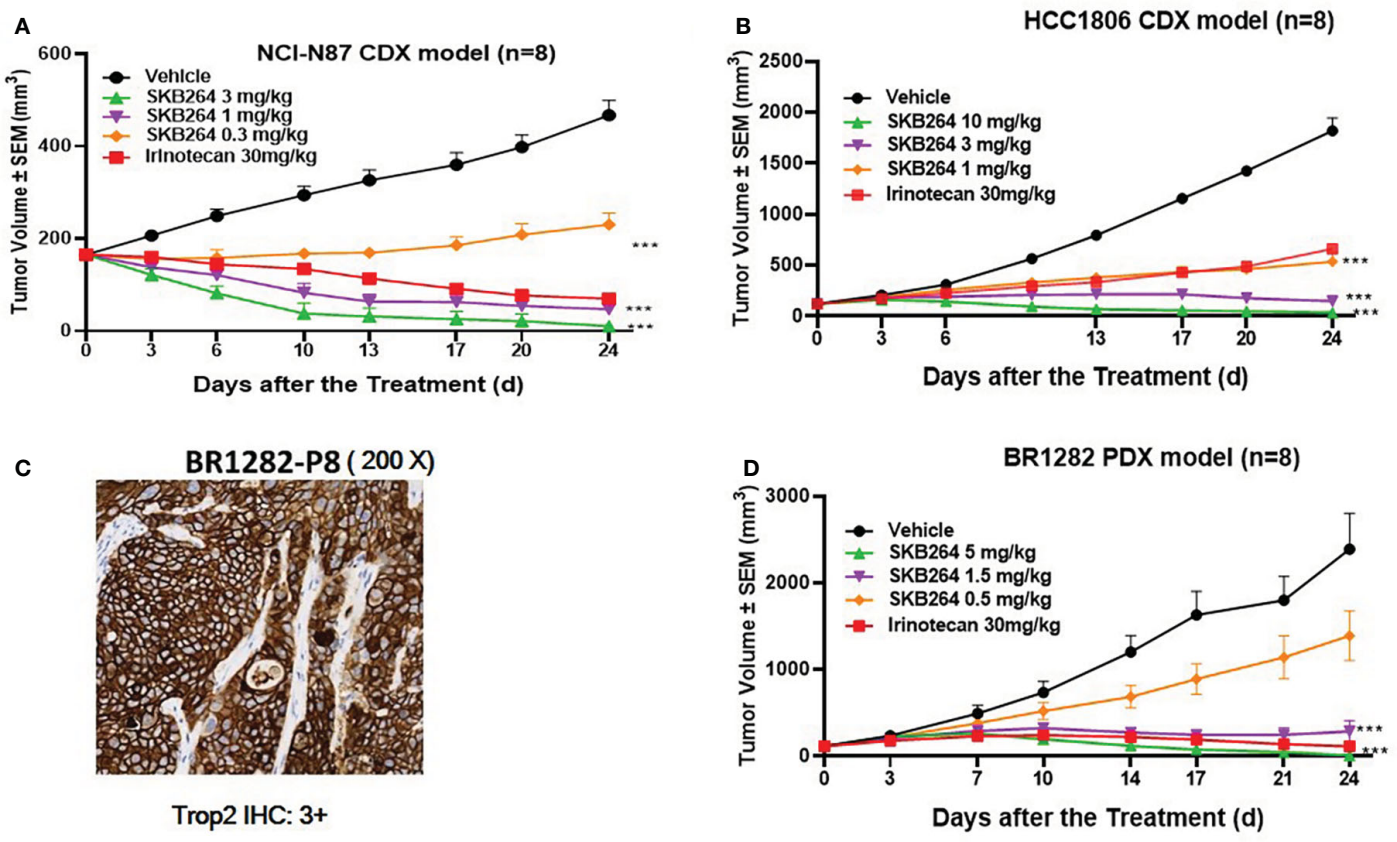
*In vivo* efficacy of the SKB264 treatment. Two-tailed paired *t*-test, ^***^ *p* < 0.001 (*vs*. vehicle). **(A)** Antitumor efficacy of SKB264 in the HCC1806 xenograft model. The tumor-bearing mice were intravenously administered with SKB264 (3, 1, and 0.3 mg/kg, respectively), irinotecan, and vehicle control, *n* = 8. **(B)** Antitumor efficacy of SKB264 in the HCC1806 xenograft model. The tumor-bearing mice were intravenously administered with SKB264 (10, 3, and 1 mg/kg) and vehicle control, *n* = 8. **(C)** IHC analysis of TROP2 expression in the BR1282 xenografted tumor. IHC staining intensity: 3+ (high expression): >10% of tumor cells showed strong cell membrane staining. **(D)** *In vivo* antitumor efficacy of SKB264 in BR1282 xenograft model (*n* = 8).

*In vivo* antitumor activity of SKB264 was evaluated using both the CDX model and the PDX model. In the xenograft tumor model of NCI-N87 cells (**Figure 4A**), on the 24th day after first dose, the percentage of TGI of SKB264 (0.3, 1, and 3 mg/kg) was 78.4%, 139.2%, and 151.2%, respectively, suggesting the high efficacy of SKB264 in the inhibition of tumor growth *in vivo* (in a dose-dependent manner, *p*-values in three dose groups were <0.001, as compared to the control group). Similar to the NCI-N87 xenograft model, in the HCC1806 xenograft model (**Figure 4B**), the percentage of TGI of SKB264 (1, 3, and 10 mg/kg) was 75.6%, 98.5%, and 105.0% (*p*-values all < 0.001), respectively, on the 24th day after first dose. Furthermore, high TROP2 expression in BR1282 tumor xenograft (derived from the tumor tissue of a breast cancer patient) was found (**Figure 4C**). As shown in **Figure 4D**, in the BR1282 PDX model, on the 24th day after the first dose, the percentage of TGI of SKB264 (0.5, 1.5, and 5 mg/kg) was 44.0%, 92.6%, and 104.8%, respectively (*p*-values in the dose groups of 1.5 and 5 mg/kg were <0.001). Overall, our study demonstrated high efficacy (in dose-dependent manner) of the *in vivo* antitumor activity of SKB264 in both CDX and PDX models.

**Figure 5:**
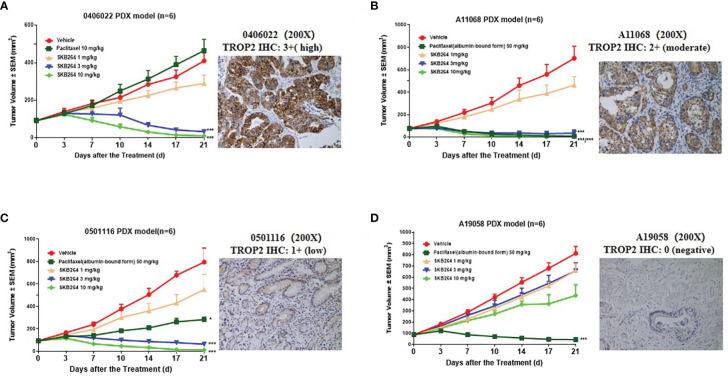
*In vivo* treatment efficacy of the SKB264 in gastric carcinoma PDX models. IHC staining intensity: 0 (Negative): no staining or ≤10% tumor cells showing any intensity of cell membrane staining; 1+ (low expression): >10% tumor cells showing weak cell membrane staining; 2+ (moderate expression): >10% tumor cells showing moderate intensity of cell membrane staining; and 3+ (high expression): >10% of tumor cells showing strong cell membrane staining. The tumor-bearing mice were intravenously treated with SKB264 (1, 3, and 10 mg/kg, respectively), vehicle control, and paclitaxel/paclitaxel (albumin-bound form) (*n* = 6 per group). On two-tailed paired *t*-test, ***p < 0.001 and *p < 0.05 (*vs*. vehicle). **(A)** 0406022; **(B)** A11068; **(C)** 0501116; and **(D)** A19058.

To explore a minimum level of TROP2 to exert an antitumoral activity, four gastric cancer PDX models with different TROP2 expression levels were transplanted into mice. As shown in **Figure 5**, the gastric cancer PDX models (0406022, A11068, 0501116, and A19058) expressed high (IHC 3+), moderate (IHC 2+), low (IHC 1+), and negative (IHC 0) levels of TROP2, respectively. SKB264 exhibited a significant antitumor effect in three TROP2-positive gastric PDX models (0406022, A11068, and 0501116), with all TGI% more than 100% at 3 mg/kg, while SKB264 did not show an antitumor effect in the TROP2-negative PDX model (A19058) at 3 mg/kg. Taken together, these results indicate that SKB264 was effective for treating TROP2-positive PDX (TROP2 IHC ≥1+). There was no significant weight loss or mortality related to treatment in any of the CDX and PDX models.

### Pharmacokinetics in cynomolgus monkeys

Firstly, the affinities of SKB264 and SKB264 mAb to TROP2 of different species were evaluated by ELISA. The EC_50_ of the affinity of SKB264 and SKB264 mAb with monkey TROP2 was 4.363 ng/ml and 3.399 ng/ml, respectively. These results correlated with the affinity of SKB264 and SKB264 mAb with human TROP2 protein. However, SKB264 and SKB264 mAb bound to rat and mouse TROP2 protein with low affinity (EC_50_ > 5,000 ng/ml), indicating that cynomolgus monkey was an appropriate species for preclinical pharmacokinetic and toxicological analysis of SKB264 because of the similar binding affinity to human TROP2. Therefore, we performed the pharmacokinetic experiments in cynomolgus monkeys.

**Figure 6:**
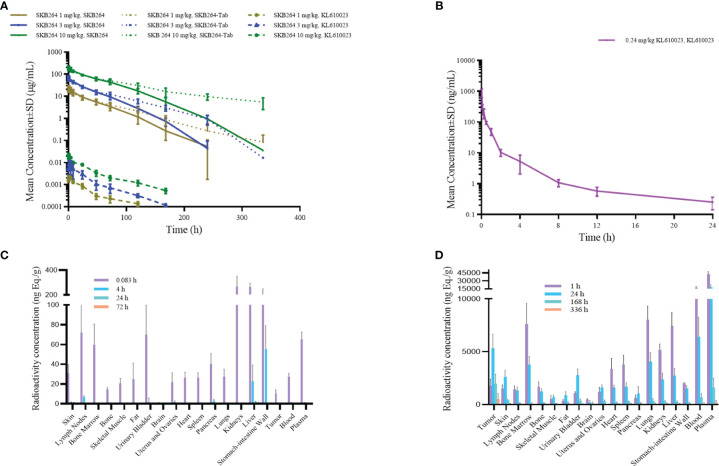
**(A)** Mean serum concentration–time curve of SKB264 (at different doses), Tab, and KL610023 following single administration (i.v.) of SKB264 in cynomolgus monkeys (*n* = 6/group). **(B)** Mean serum concentration–time curve of KL610023 following single administration (i.v.) of KL610023 in cynomolgus monkeys (*n* = 6). **(C)** Total radioactivity (TRA) in blood, plasma, and normal tissues following a single intravenous dose of [^3^H]KL610023 at 0.1 mg/100 µCi/kg in xenografted nude mice (*n* = 6). **(D)** TRA in blood, plasma, and normal tissues following a single intravenous dose of [^3^H-KL610023]SKB264 at 3 mg/100 µCi/kg in xenografted nude mice (*n* = 6).

The effect of the increased exposure of SKB264, Tab, and KL610023 in cynomolgus monkeys was roughly proportional to that of the increasing dose of a single intravenous administration of SKB264 (1 mg/kg, 3 mg/kg, and 10 mg/kg, respectively). The clearance of SKB264 was slow after a single injection (*t*_1/2_: 24–57 h) (**Figure 6A**; **Table 2**), while the KL610023 was cleared rapidly (*t*_1/2_: 5.7 h) after a single intravenous administration at a dose of 0.24 mg/kg (**Figure 6B**; **Table 3**), indicating that SKB264 can significantly prolong the half-life of the payload (KL610023). Although KL610023 would be released in the plasma following the injection of SKB264, it accounted for <0.010% of systemic exposure of SKB264, which benefited from the unique design of SKB264 to ensure its stability *in vivo*.

**Table 2:** Serum pharmacokinetic parameters of ADC and Tab after administration of SKB264 in cynomolgus monkeys (mean ± SD).

Parameters	1 mg/kg		3 mg/kg		10 mg/kg	
	ADC	Tab	ADC	Tab	ADC	Tab
*t*_1/2_ (h)	23.8 ± 3.92	35.3 ± 12.6	24.8 ± 2.99	41.0 ± 8.40	25.2 ± 4.36	56.5 ± 17.5
*T*_max_ (h)	0.178 ± 0.249	0.342 ± 0.400	0.261 ± 0.410	0.506 ± 0.440	0.425 ± 0.483	0.670 ± 0.403
*C*_max_ (µg/ml)	25.2 ± 6.03	21.4 ± 5.04	77.4 ± 12.0	65.3 ± 6.64	214 ± 24.4	189 ± 26.7
AUC_0-t_ (h·µg/ml)	787 ± 216	871 ± 252	2,170 ± 349	2,370 ± 404	8,550 ± 1,160	10,200 ± 2,210
AUC_0-∞_ (h·µg/ml)	792 ± 214	879 ± 249	2,250 ± 305	2,570 ± 401	8,640 ± 1,070	10,800 ± 2,050

**Table 3:** Serum pharmacokinetic parameters of the payload after administration of SKB264 and KL610023 in cynomolgus monkeys (mean ± SD).

Parameters	SKB264 group			KL610023 group
	1 mg/kg (*n* = 6)	3 mg/kg (*n* = 6)	10 mg/kg (*n* = 6)	0.24 mg/kg (*n* = 6)
*t*_1/2_ (h)	36.3 ± 17.7	43.8 ± 15.0	37.8 ± 10.8	5.65 ± 2.53
*T*_max_ (h)	1.50 ± 1.22	1.25 ± 1.40	0.345 ± 0.508	NA
*C*_max_ (ng/ml)	2.29 ± 0.365	8.84 ± 3.95	23.8 ± 2.09	NA
AUC_0-t_ (h*ng/ml)	57.9 ± 14.5	218 ± 106	599 ± 90.2	228 ± 44.2
AUC_0-∞_ (h*ng/ml)	66.0 ± 17.4	232 ± 102	622 ± 80.9	230 ± 43.9

NA: Not applicable.

### Tissue distribution of SKB264 in tumor-bearing mice

After a single intravenous administration of [^3^H-mAb]SKB264 or [^3^H]SKB264-mAb in the tumor-bearing mice, the distribution and elimination trends of total radioactivity of the isotope-labeled mAb or ADC were similar, indicating that the targeting and distribution behavior of SKB264 mAb remained without remarkable change after attaching payload. After a single intravenous administration of the payload, i.e., 0.1 mg/100 µCi/kg [^3^H]KL610023, its radioactivity was widely distributed in the tissues and organs that had more abundant blood flow (such as lymph nodes, bone marrow, plasma, intestine, stomach, lung, liver, and kidney) and would be quickly cleared (**Figure 6C**). However, the relative abundance of drug was higher in tumor tissue after administration of [^3^H]KL610023]SKB264, compared with [^3^H]KL610023 (**Figure 6D**). The elimination of total radioactivity in tumor tissue was also slower. These results indicate that SKB264 has abilities of target-specific uptake and retention in the tumors with high TROP2 expression.

### Safety profile of SKB264

The *in vivo* safety study was performed in cynomolgus monkeys, in which the repeated intravenous dosing of SKB264 (every 2 weeks for four doses) was conducted. In detail, 40 cynomolgus monkeys were randomized into four groups to receive saline or different doses of SKB264 (25, 50, and 75 mg/kg) *via* i.v. infusion. The first dosing day was defined as Day 1. Five monkeys (two females and three males) in the group of SKB264 (75 mg/kg) treatment were euthanized due to their status of moribundity on Days 10–24. The cause of the moribundity appeared to be the deteriorating condition of the animal, the symptoms of which included loss of body weight, reduced food consumption, bone marrow toxicity, and intestinal toxicity. Histopathological examination found several abnormalities in in the group of SKB264 (75 mg/kg), including intestinal villi atrophy, chronic inflammation, intestinal gland dilatation, thymic cortex lymphopenia and cysts, bone marrow hematopoietic cell reduction, skin hair follicle atrophy, pigmentation and other morbid conditions, and changes in the corresponding clinic pathology indicators. In the SKB264 (50 mg/kg) group, there were also similar changes, but its incidence or severity was less or low. However, in the SKB264 (25 mg/kg) group, only the lesions in the skin and thymus were observed, most of which could be recovered or unobserved at the end of the recovery phase, except for the lesions in thymus. Although gastrointestinal toxicity and bone marrow toxicity are typical dose-limiting factors related to the clinical application of topoisomerase I inhibitors, in our study, the observed SKB264 effect on the intestines was very slight, even in the SKB264 (50 mg/kg) group. The dramatic decrease of side effects was presumable due to less off-target toxicity. TROP2-negative cells and the PDX model were less sensitive to SKB264, probably because it mainly targets and releases payload in TROP2-positive cells or tissues (**Figures 2D**, **5**). The bone marrow toxicity, accompanied by a decreased reticulocyte ratio, was observed only in the SKB264 (50 mg/kg) group. In the thymus of monkeys in the SKB264 (25 mg/kg) group, the decreased cortical lymphocytes had no toxicological significance as shown in **Table 1**. No accumulation was found after the four repeated dosing of SKB264 (75 mg/kg). There was no apparent gender difference of the AUC and *C*_max_ of SKB264 after the first dosing in the SKB264 (75 mg/kg) group, but they were higher in females than the males after the last dosing. Under the experimental conditions, the highest non-seriously toxic dose (HNSTD) of SKB264 was 50 mg/kg in monkeys following the repeated administration. The SKB264 HNSTD in monkeys provides evidence of preclinical safety of this ADC, which meets the requirement of further clinical trial. Therefore, those data are acceptable for entry into human clinical trials.

### Comparison of the efficacy between SKB264 and IMMU-132

**Figure 7:**
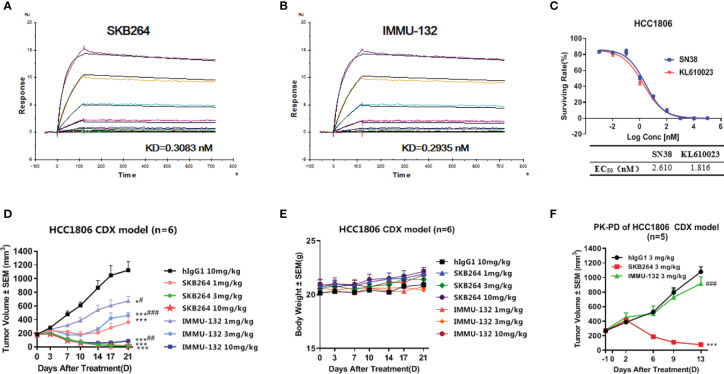
**(A)** Dynamic affinity of SKB264 to human TROP2. **(B)** Dynamic affinity of IMMU-132 to human TROP2. **(C)** *In vitro* inhibitory effect of KL610023 and SN-38 on HCC1806 cell growth (duplicates, mean ± SEM). **(D)** Antitumor efficacy of SKB264 and IMMU-132 in the HCC1806 xenograft model. The tumor-bearing mice were intravenously treated with SKB264 or IMMU-132 at dosages of 10, 3, and 1 mg/kg twice weekly, *n* = 6. Two-tailed paired *t*-test, ^*^ *p* < 0.05; ^***^ *p* < 0.001 (*vs*. hIgG1); ^#^ *p* < 0.05; ^##^ *p* < 0.01; ^###^ *p* < 0.001 (*vs*. the same dose of SKB264). **(E)** Body weight of the tumor-bearing mice (*n* = 6). **(F)** Antitumor efficacy of single-dose SKB264 and IMMU-132 in the HCC1806 xenograft model in PK-PD (*n* = 5). Two-tailed paired *t*-test, ^***^ *p* < 0.001 (*vs*. hIgG1); ^###^ *p* < 0.001 (*vs*. SKB264).

Both SKB264 and IMMU-132 are the TROP2-targeting ADCs, and their antibodies share the same amino acid sequence. SPR study revealed that both SKB264 and IMMU-132 could bind to the recombinant human TROP2 with *K*_d_ values of 0.3083 nM and 0.2935 nM, respectively, demonstrating that SKB264 and IMMU-132 have similar affinity for TROP2 (**Figures 7A, B**). Although the antibodies are similar, the coupled payload of SKB264 is KL610023, a derivative of belotecan, while that of IMMU-132 is SN-38, an active metabolite of irinotecan. Nevertheless, those two coupled payloads were potent to inhibit the proliferation of HCC1806 cells; as shown in **Figure 7C**, the IC_50_ of KL610023 and SN-38 was 1.816 nM and 2.610 nM, respectively. An *in vitro* study showed that there was no statistically significant difference in pharmacological activity between those two payloads. However, the difference in *in vivo* efficacy between SKB264 and IMMU-132 was found in our study, which might be due to their different linker and payload. In the HCC1806 xenograft tumor model, SKB264 could significantly inhibit tumor growth at doses of 1, 3, and 10 mg/kg, and the TGI was 81.06%, 189.46%, and 188.86%, respectively. In the same xenograft tumor model, the TGI of the IMMU-132 treatment (1, 3, and 10 mg/kg) was 48.12%, 70.64%, and 153.27%, respectively (**Figure 7D**). On the other hand, there was no significant change in the body weight of the experimental mice in all treatment groups (**Figure 7E**), indicating that SKB264 had a stronger ability to inhibit tumor growth than IMMU-132 at the same dose, so that the low dose of SKB264 could reach the same efficacy of the high dose of IMMU-132, avoiding stronger adverse reaction.

The PK-PD study using the HCC1806 xenograft tumor model showed that the TGI of SKB264 (3 mg/kg) or IMMU-132 (3 mg/kg) after a 13-day single administration was 123.47% and 21.05%, respectively, indicating that the inhibitory effect of SKB264 on tumor growth was stronger than that of IMMU-132 after a single administration at the same dose (**Figure 7F**). Meanwhile, a PK study also found that the mouse plasma payload exposure of both SKB264 and IMMU-132 was similar, but the intratumor payload exposure of SKB264 was about 4.6 times that of IMMU-132 (**Table 4**). The larger intratumor exposure of SKB264 accounted for the better TGI, implying its greater effectiveness in cancer treatment.

**Table 4:** Payload pharmacokinetic parameters in tumor and plasma after administration of either SKB264 or IMMU-132 in tumor-bearing mice (mean, *n* = 5).

Measured object	Tumor		Plasma	
	KL610023	SN-38	KL610023	SN-38
*t*_1/2_ (h)	52.8	57.4	56.3	15.5
*T*_max_ (h)	96	24	1	1
*C*_max_ (ng/g or ng/ml)	14.6	4.5	0.53	1.32
AUC_last_ (h*ng/g or h*ng/ml)	1903	410	22.94	19.26
AUC_0-∞_ (h*ng/g or h*ng/ml)	1949	448	32.68	22.17

Remarks: KL610023 is the payload of SKB264, while SN-38 is the payload of IMMU-132.

### *In vitro* stability of SKB264 and IMMU-132 in plasma

**Figure 8:**
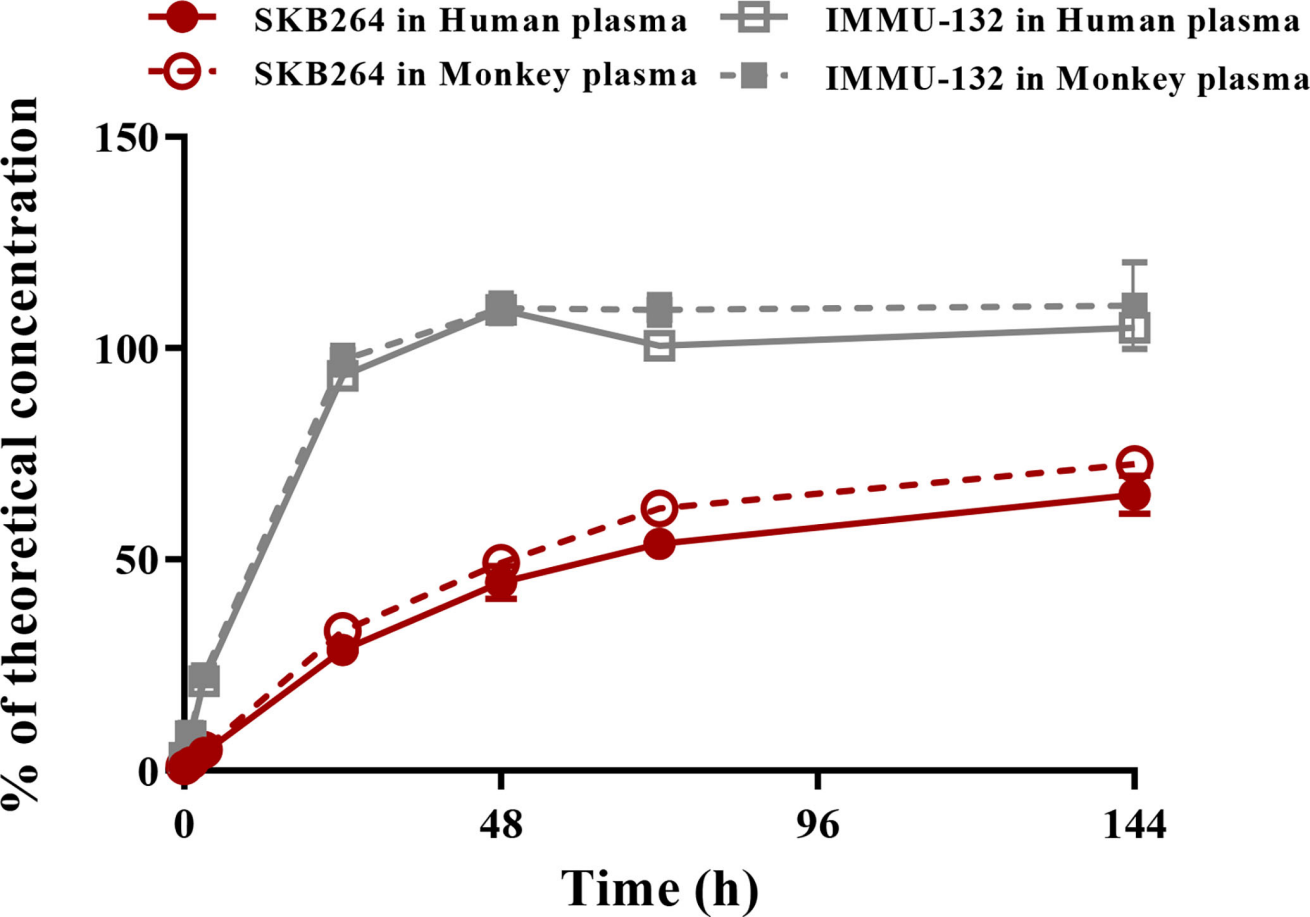
Percent of free payload in total available payload during the incubation of SKB264 or IMMU-132 with plasma of monkey and human.

The release rates of the payload from IMMU-132 in cynomolgus monkeys and human plasma were almost 100% after 48-h incubation, while only 70% payload was released from SKB264 after 144-h incubation (**Figure 8**). These results indicate that SKB264 is more stable and releases payload more slowly in cynomolgus monkeys and human plasma than IMMU-132.

## Discussion

**Figure 9:**
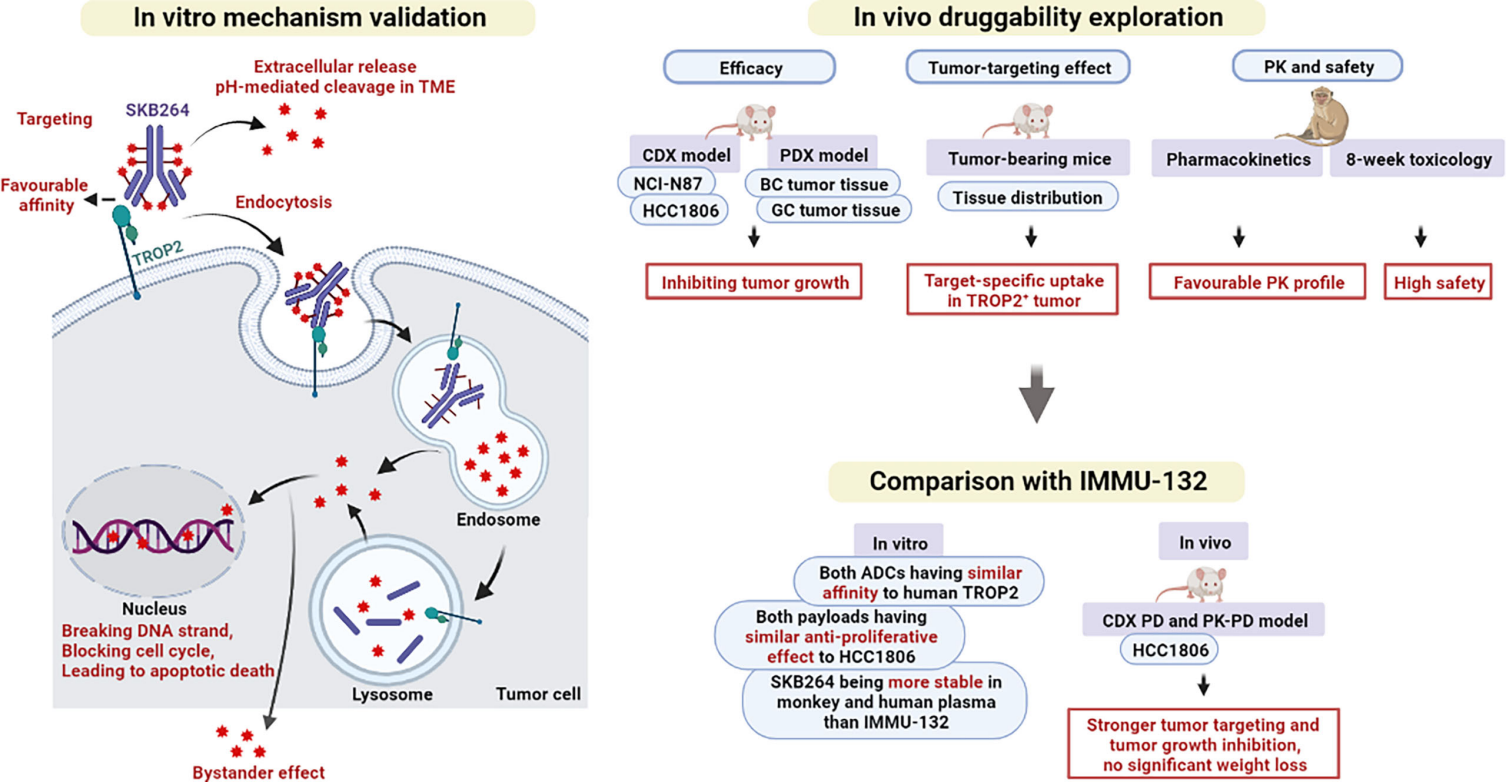
Graphical summary of the main findings in this study.

Cancer is characterized by the uncontrollable growth of abnormal cells, causing the disease that seriously affects human health and quality of life (18). It is reported that about one-sixth of all deaths are caused by cancer (19). TROP2 is a protein that is highly expressed in many epithelial tumors, including breast, lung, gastric, colorectal, pancreatic, prostatic, cervical, head-and-neck, and ovarian carcinomas, making it a potential and important target for ADCs (20, 21). As shown in **Figure 9**, based on the specificity of mAbs to recognize the cell-surface antigens of cancer, ADCs can deliver toxic payload to the tumor site, thereby enhancing the cytotoxicity in the tumor cells and reducing the toxic effect of drug in normal tissues (8). In April 2020, sacituzumab govitecan (IMMU-132), the first anti-TROP2 ADC, was approved in the US for the treatment of metastatic TNBC (22), spurring more research on the TROP2 biology and the development of anti-TROP2 ADC. Moreover, IMMU-132 was also used to treat advanced solid tumors in clinical studies (23).

Since TROP2 is also expressed in multiple normal tissues, concerns have been raised on the specificity of anti-TROP2 ADC (1). Anti-TROP2-ADC may produce strong systemic toxicity in normal organs before reaching the effective level. Like any other treatment method, the therapeutic window is the key factor to tap the potential of ADC drugs. It has been reported that IMMU-132 development is successful because of lower payload toxicity than other ADCs, such as T-DM1 (1). However, IMMU-132 is not perfect, because the breakage of the linker–payload conjugate from this ADC may occur through thiol exchange under physiological conditions, making it relatively unstable in the circulation (24). The shedding of payload (SN-38) in the circulatory system will cause off-target toxicity, such as neutropenia, diarrhea, vomiting, and nausea (FDA Blackbox Warning) (25, 26). To minimize the off-target toxicity of ADCs like IMMU-132, SKB264 is developed with a novel DNA topoisomerase I inhibitor (KL610023) and a linker that confers an optimized conjugation method. Unlike the reversible addition of maleimide to cysteine in IMMU-132, methylsulfonyl pyrimidine is coupled with the linker of SKB264, *via* an irreversible covalent binding with disulfide reduced cysteine, by which the problem that the payload falls off easily from ADC can be fixed. Moreover, the use of toxic payload KL610023 further increases the stability of SKB264 as well. We hope to enhance the targeting ability of SKB264, reduce its off-target toxicity, and obtain a broader therapeutic window through the innovative design of SKB264.

To verify the targeting and antitumor effects of SKB264, both *in vivo* and *in vitro* experiments were conducted in the present study (**Figure 9**). The results of comparison showed that there was no significant difference between SKB264 and SKB264 mAb, in respect of their affinity (*in vitro*) to TROP2 protein and their ability to bind to TROP2 expressed on HCC1806 and NCI-N87 cells, followed by being internalized, suggesting that KL610023 conjugation cannot affect the affinity of SKB264 mAb. On the other hand, the ability of tumor targeting and internalization of SKB264 was mediated by SKB264 mAb. Furthermore, the results of cytotoxic assay showed that the antitumor effect of either SKB264 or KL610023 was almost the same, but the SKB264 mAb did not affect the survival of tumor cells, suggesting that KL610023 is the main cytotoxic factor to kill tumor cells. In *in vivo* experiments, SKB264 could significantly improve the TGI rate in both CDX and PDX models in a dose-dependent manner and also manifest better antitumor effect *in vivo*, as compared to that of IMMU-132 at the same dose. As to the mechanism of its action, SKB264 targets TROP2-positive tumor cells and mediates endocytosis *via* SKB264 mAb. After internalization, SKB264 releases its payload (KL610023) to kill tumor cells. However, there is variation in the process of SKB264 internalization and KL610023 release, which might be due to the difference in the factors related to intracellular trafficking pathways, for example, clathrin- or caveolin-mediated endocytosis, or lysosomal enzyme activities (27). Recycling and turnover of the TROP2 protein could also affect the KL610023 release rate (28). Therefore, further analysis is necessary to clarify the detailed dynamics of SKB264 in tumor cells.

PK experiments for SKB264 were conducted in cynomolgus monkeys, and the results showed that the affinity of both SKB264 and SKB264 mAb to TROP2 protein of cynomolgus monkeys was similar to that of human, suggesting that the cynomolgus monkey is the most appropriate and related species to human for the preclinical study of SKB264. We found that the concentrations of SKB264, Tab, and KL610023 in the serum of cynomolgus monkeys were roughly proportional to the dosage after a single intravenous administration, indicating that their metabolic process was stable *in vivo* (29). Since the metabolism of ADC is different from that of the toxic payload (30, 31), the linkage of SKB264 mAb can dramatically prolong the half-life of KL610023 as compared to KL610023 alone. Nevertheless, after being released from SKB264, KL610023 could be rapidly eliminated. Its proven short half-life (1.6 h) in cynomolgus monkeys leads to less toxicity or side reactions in normal tissues (**Table 5**) (32). In addition, only a low level of KL610023 could be detected after a single intravenous administration of SKB264, suggesting the high stability of SKB264. These results indicate that the modification of SKB264 mAb can reduce the exposure of KL610023 in the circulatory system, and the drug-mediated damage in normal tissues could be significantly reduced (33). Moreover, the isotope labeling experiments revealed that the linkage of SKB264 mAb was associated with a slow clearance of KL610023 and showed its enrichment in the tumor tissues, suggesting that this linkage may effectively improve the antitumor effect of KL610023. Overall, the proprietary technology used in our study to construct SKB264 could ensure a more precise delivery process.

**Table 5:** Payload KL610023 pharmacokinetic parameters in plasma after administrated in cynomolgus monkey (n=4, half male and half female).

Measured object	KL610023
t_1/2_ (h)	1.61 ± 1.16
C_max_ (ng/mL)	273 ± 54.4
AUC_last_ (h*ng/mL)	97 ± 26.7
AUC_0-∞_ (h*ng/mL)	97 ± 26.9
V_z_obs_ (h·ng/mL)	1119 ± 548
Cl__obs_ (mL/kg)	535 ± 148

Remarks: KL610023 is the payload of SKB264.

Similar to IMMU-132 (10), the off-target toxicity of SKB264 observed in this study mainly occurred in the TROP2 highly expressed tissues (34), including the gastrointestinal, hematopoietic, and immune systems. In addition, the metabolic related experiments showed that unlike the metabolism of SN-38, KL6100023 had no hepatic circulation. In contrast, SN-38G can be converted into SN-38 in the intestine *via* its hepatic circulation (35). Therefore, as compared to IMMU-132, KL6100023 has less gastrointestinal adverse reactions, such as diarrhea (11). Moreover, within the dose range of 25–75 mg/kg, there was no significant gender difference in respect of the exposure/concentration of SKB264 in monkey serum; thus, the increased serum concentration of SKB264 was basically proportional to dose escalation. Under the experimental conditions, the HNSTD of SKB264 was 50 mg/kg, which was far greater than the pharmacodynamics dose of the model mice (3 mg/kg), indicating that the treatment window of SKB264 was wide enough, because of its stable linker leading to less payload release in circulation. This advantage enables less hematotoxicity and gastrointestinal toxicity, providing the opportunity to a possible combination therapy of SKB264.

It is noteworthy that there was difference in the *in vivo* behavior between SKB264 and IMMU-132, due to the features of their different linkers. The release rate of the payload of IMMU-132 was almost 100% in the plasma of both cynomolgus monkey and human, after 48-h incubation, while only 70% payload was released from SKB264 after 144-h incubation. The payload releasing curve of IMMU-132 was closer to the curve of the exponential growth before reaching the peak, while that of SKB264 was closer to the curve of the linear growth, indicating that SKB264 was more stable in plasma than IMMU-132. Furthermore, in case of the same dose, the payload exposure of SKB264 in tumor tissue is about 4.6 times that of IMMU-132 found in the PK-PD study. The intratumoral accumulation of KL6100023 is associated with the better stability of SKB264 in plasma and its longer circulation time *in vivo* (11). Taken together, the findings of our study can explain why the anticancer effect of SKB264 is better than that of IMMU-132 at the same dose.

The potentially lower hematotoxicity and gastrointestinal toxicity feature of SKB264 might provide the opportunity of a possible combination therapy with other drug modalities. For example, the combination therapy with both Topo I inhibitor and poly(ADP-ribose) polymerase (PARP) inhibitor is an attractive therapeutic approach, because both of them target the DNA damage response (DDR) pathway with promising results from preclinical studies (36, 37). However, this kind of combination therapy is still challenged by the overlap of the hematological side effects (38–40). Relying on its unique feature of less myelosuppressive side effects, SKB264 could offer improved clinical outcomes in the combination therapy with PARP inhibitors. Another attractive feature of the combination therapy with SKB264 might be immunotherapy, because Topo I inhibitors can improve the antitumor effects of immune checkpoint inhibitors (ICIs) (41–43). In the preclinical syngeneic mouse tumor model, it has been demonstrated that trastuzumab deruxtecan could sensitize tumors to ICI therapy by the enhanced antitumor immunity, which is activated by the delivered DNA topoisomerase I inhibitor payload (44–46). Therefore, the similar combination therapy with SKB264 and ICIs is expected and its preclinical evaluation is in progress.

In summary, we developed SKB264, a novel TROP2-targeting ADC using several patented technologies. Our study showed the remarkable efficacy of SKB264 in the nonclinical TROP2-expressing xenograft models, with an acceptable safety profile and an excellent therapeutic window in animal studies, suggesting that SKB264 treatment may be a promising therapeutic therapy for treating gastric carcinoma and breast cancer in the clinical setting. With its highly promising results in preclinical studies, SKB264 has been applied in clinical trials, hopefully to provide clinical benefit for patients with gastric carcinoma, lung cancer, and breast cancer.

## Data availability statement

The raw data supporting the conclusions of this article will be made available by the authors, without undue reservation.

## Ethics statement

The animal study was reviewed and approved by Institutional Animal Care and Use Committee (IACUC).

## Author contributions

YZC: Conceptualization, formal analysis, investigation, methodology, and writing—original draft. XY, QT, XH and YC: Conceptualization, methodology, and writing—original draft. YP and HL: Formal analysis, methodology, and writing—review and editing. MX, YJ, JX and YT: Investigation and writing—review and editing. XZ and HS: Project management and writing—review and editing. All authors contributed to the article and approved the submitted version.

## Glossary

**Table:**

ADC	antibody–drug conjugate
Tab	total antibody
SKB264 mAb	monoclonal antibody (mAb) of SKB264
ADA	anti-drug antibody
AUC_0-t_	area under curve from 0 to t
AUC_0-∞_	area under curve from 0 to infinity
CDX	cell-derived xenograft
DAR	drug-to-antibody ratio
HNSTD	highest non-seriously toxic dose
HGB	hemoglobin
HCT	hematocrit
IL-2	interleukin-2
IL-6	interleukin-6
IL-8	interleukin-8
IFN-γ	interferon-gamma
ICIs	immune checkpoint inhibitors
LYM	lymphocytes
mAb	monoclonal antibody
*C*_max_	maximal plasma concentration
NEU	neutrophils
ORR	objective response rate
PK-PD	pharmacokinetics–pharmacodynamics
PDX	patient-derived xenograft
PARP	poly(ADP-ribose) polymerase
RBC	red blood cell count
RET	reticulocyte percentage
TROP2	trophoblast antigen 2
TACSTD2	tumor-associated calcium signal transductor
TNBC	triple-negative breast cancer
TNF-α	tumor necrosis factor-alpha
TK	toxicokinetics
TGI	tumor growth inhibition
*t*_1/2_	half-life
*T*_max_	time to maximal plasma concentration
WBC	white blood cells
