# Corrigendum: Preclinical profiles of SKB264, a novel anti-TROP2 antibody conjugated to topoisomerase inhibitor, demonstrated promising antitumor efficacy compared to IMMU-132

**DOI:** 10.3389/fonc.2023.1334938

**Published:** 2023-12-07

**Authors:** Yezhe Cheng, Xiaoxi Yuan, Qiang Tian, Xiuying Huang, Yang Chen, Yuzhi Pu, Hu Long, Mingyu Xu, Yafei Ji, Jia Xie, Yuping Tan, Xi Zhao, Hongmei Song

**Affiliations:** Center of Translational Medicine, Sichuan Kelun-Biotech Biopharmaceutical Co., Ltd., Chengdu, China

**Keywords:** antibody drug conjugate, SKB264, TROP2, PK-PD, solid tumor

**Error in Table**

In the published article, there was an error in **Table 5** as published.

The corrected **Table 5** and its caption “Payload KL610023 pharmacokinetic parameters in plasma after administrated in cynomolgus monkey (n=4, half male and half female)” appears below.

**Table 5:** Payload KL610023 pharmacokinetic parameters in plasma after administrated in cynomolgus monkey (n=4, half male and half female).

Measured object	KL610023
t_1/2_ (h)	1.61 ± 1.16
C_max_ (ng/mL)	273 ± 54.4
AUC_last_ (h*ng/mL)	97 ± 26.7
AUC_0-∞_ (h*ng/mL)	97 ± 26.9
V_z_obs_ (h·ng/mL)	1119 ± 548
Cl__obs_ (mL/kg)	535 ± 148

Remarks: KL610023 is the payload of SKB264.

The authors apologize for this error and state that this does not change the scientific conclusions of the article in any way. The original article has been updated.

